# Computing Complex Visual Features with Retinal Spike Times

**DOI:** 10.1371/journal.pone.0053063

**Published:** 2013-01-02

**Authors:** Robert Gütig, Tim Gollisch, Haim Sompolinsky, Markus Meister

**Affiliations:** 1 Max Planck Institute of Experimental Medicine, Göttingen, Germany; 2 Racah Institute of Physics and Interdisciplinary Center for Neural Computation, Hebrew University, Jerusalem, Israel; 3 University Medical Center Göttingen, Department of Ophthalmology, Göttingen, Germany; 4 Center for Brain Science, Harvard University, Cambridge, Massachusetts, United States of America; 5 Department of Molecular and Cellular Biology, Harvard University, Cambridge, Massachusetts, United States of America; Oregon Health & Science University, United States of America

## Abstract

Neurons in sensory systems can represent information not only by their firing rate, but also by the precise timing of individual spikes. For example, certain retinal ganglion cells, first identified in the salamander, encode the spatial structure of a new image by their first-spike latencies. Here we explore how this temporal code can be used by downstream neural circuits for computing complex features of the image that are not available from the signals of individual ganglion cells. To this end, we feed the experimentally observed spike trains from a population of retinal ganglion cells to an integrate-and-fire model of post-synaptic integration. The synaptic weights of this integration are tuned according to the recently introduced tempotron learning rule. We find that this model neuron can perform complex visual detection tasks in a single synaptic stage that would require multiple stages for neurons operating instead on neural spike counts. Furthermore, the model computes rapidly, using only a single spike per afferent, and can signal its decision in turn by just a single spike. Extending these analyses to large ensembles of simulated retinal signals, we show that the model can detect the orientation of a visual pattern independent of its phase, an operation thought to be one of the primitives in early visual processing. We analyze how these computations work and compare the performance of this model to other schemes for reading out spike-timing information. These results demonstrate that the retina formats spatial information into temporal spike sequences in a way that favors computation in the time domain. Moreover, complex image analysis can be achieved already by a simple integrate-and-fire model neuron, emphasizing the power and plausibility of rapid neural computing with spike times.

## Introduction

In most of the vertebrate nervous system, neurons communicate by all-or-nothing action potentials rather than graded potentials. It is commonly assumed that neurons transmit information using their average firing rate, namely by modulating the number of spikes produced in a coarse window of time or in a large neuronal population [1]. Indeed, the characterization of spike trains by their mean firing rates has been the dominant approach in the vast majority of electrophysiological and computational modeling studies.

Several observations have already challenged the rate-based description of neuronal processing and stoked interest in temporal neural codes that involve the timing of single spikes. Studies of the visual, auditory, olfactory, and somatosensory pathways have revealed precise timing relationships in neuronal firing patterns elicited by sensory stimuli [2]–[7], suggesting that an important component of stimulus information could be encoded in the timing of individual spikes [8].

In the visual system, spike timing codes may be particularly relevant in the context of the natural dynamics of vision. In humans and most other animals, vision occurs in discrete episodes where the eye is relatively still, interrupted by rapid gaze shifts called “saccades”. During such a saccade, the visual image sweeps rapidly over the retina, and several retinal ganglion cell types are strongly suppressed [9]. After the image comes to rest, many ganglion cells fire a burst of spikes [10]–[12]. These bursts of spikes during the fixation period comprise all the retinal information available for processing the new scene.

Recently, it has been shown that certain ganglion cells of the salamander retina encode information about the spatial content of a newly encountered image in the timing of the very first spike after image onset [13]. Based on spike times measured in populations of such retinal ganglion cells, we here explore how a neuronal readout model can use this information to compute image information that is not available from responses of individual ganglion cells. To do so, we employ the simplest of downstream neural circuits: a single post-synaptic neuron with suitably adjusted synaptic weights for its afferents. By optimizing the weights according to the recently introduced tempotron learning rule [14], we test whether the readout neuron can detect predefined classes of visual stimuli by spiking in response while remaining silent for other stimuli. Despite the simplicity of this model, we find that it can already perform surprisingly sophisticated visual computations on the received retinal signals: It can detect specific stimulus features while remaining invariant to the polarity and strength of the image contrast. Building on the preceding reports [13], [14], we now show that the temporal code generated by the retina is, in fact, highly conducive to the temporal computations performed by the integrate-and-fire neuron. It will be seen that both selectivity and invariance of important visual detection tasks emerge almost trivially once they are formatted in the time domain.

## Results

### Certain Retinal Ganglion Cells Encode a New Image by Spike Latencies

**Figure 1:**
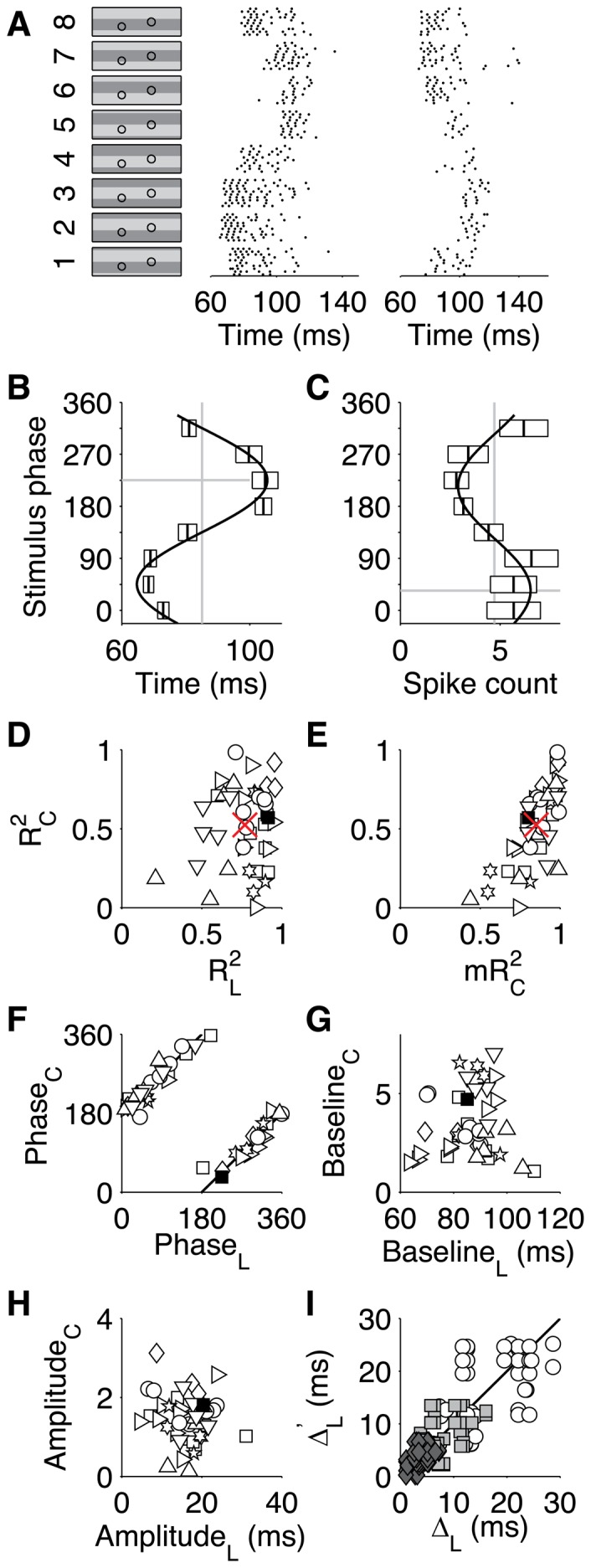
Tuning of retinal ganglion cell responses. (**A**) Illustration of retinal ganglion cell recordings used in this work. Left, the eight grating patterns used as stimuli, along with the receptive field centers of two recorded fast-Off RGCs. One grating period measured 660 µm on the retina. Middle and right, spike rasters recorded from these two RGCs, plotted vs time since the onset of each grating stimulus. The gratings were presented multiple times randomly interleaved. (**B**) Cosine fit (solid line) to the first-spike latencies of the RGC from (**A**), middle column. Boxes depict mean latency ±1SD. Gray lines show the baseline (vertical) and phase offset (horizontal) of the cosine fit. (**C**) As in (**B**), but for the spike count. (**D**) Quality of the cosine fits, measured by coefficients of determination for single-trial first-spike latencies, 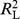, and of single-trial spike counts, 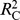, in the highest contrast condition. Each point is one RGC, different symbols denote different experiments, the filled square represents the fits shown in (**B**) and (**C**). The cross marks the population means. (**E**) As in (**D**), but coefficients of determination for single-trial spike counts, 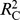, plotted versus each fit’s coefficients of determination for mean spike counts, 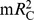. The 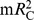 values are large even when the corresponding 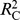 is small, which indicates that 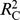 is affected by large noise in the spike counts rather than a systematic failure of the cosine model. (**F**) Scatter plot of phase offsets for the cosine fits of spike count (y-axis) and latency (x-axis). The solid black line depicts a relative phase shift of 180°. (**G**) As in (**F**), but for the baseline values. (**H**) As in (**F**), but for modulation amplitudes. (**I**) Contrast affects response latency similarly in different cells. For each RGC, cosine fits for the latency were obtained at all four studied contrast levels and the shift 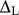 of the latency baseline was measured relative to the highest contrast, i.e. from 47% to 39% (diamonds), 47% to 31% (squares), and 47% to 29% (circles). For all pairs of RGCs analyzed, this scatterplot shows the baseline shifts of the two members. Solid line is the identity.

We analyzed spiking responses of retinal ganglion cells (RGCs) in the salamander retina to the appearance of a new image on the retina. The stimulus was a uniform gray field followed by a square grating [13]. We presented the grating at eight different spatial phases. A micro-electrode array recorded spike trains simultaneously from many retinal ganglion cells. The RGC population consists of several types, and we focus here on the so-called “fast-Off” cells [13], [15]. These neurons exhibit low or zero baseline activity and generally fire in response to both an increase and decrease in intensity on the receptive field. To the grating stimuli in the present study, they typically responded with a burst of spikes, regardless of the position of the grating [13]. However, the latency of the spike burst varied systematically with the grating position (Figure 1A).

To better understand how the stimulus identity is represented by these neurons, we inspected more closely the tuning curves for two parameters of the spike burst: the latency, namely the time of the first spike following stimulus onset; and the total spike count in the response interval. Both the latency and the spike count depended on grating phase in approximately sinusoidal fashion (Figures 1B, C). For the 41 fast-Off RGCs analyzed in the present study, both the *mean* latencies and the *mean* spike counts were well fitted by cosine tuning curves (Figures 1D, E; coefficients of determination 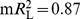 and 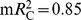, respectively). On the *single-trial* level, however, only the first-spike latencies were faithfully described by cosine tuning (average 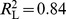), whereas the tuning fidelity for spike counts was substantially lower (average 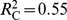), reflecting a high trial-to-trial variability in the spike counts. These tuning characteristics are consistent with a prior report that latencies typically convey more stimulus information than spike counts for this cell type [13].

Each of these tuning curves had a different phase offset, depending on the location of the corresponding cell’s receptive field center, and the population covered all phase offsets roughly uniformly (Figure 1F). The tuning curves for latency and spike count were shifted by ∼180° (Figure 1F), consistent with the expectation that strong stimuli elicit short latencies and high spike counts. Interestingly, neither the baseline values (Figure 1G) nor the modulation amplitudes (Figure 1H) of the cells’ first-spike latencies appeared to be correlated with the corresponding parameters of the spike count tuning curves, indicating that the characteristics of latency coding and spike-count coding are independently distributed within this ganglion cell class.

An increase in stimulus contrast generally produced a decrease in latency (Figure 1I) and an increase in spike count (not shown). These shifts were mostly additive, affecting all eight stimuli in a similar fashion. Thus contrast affected mostly the baselines of the tuning curves, not their amplitude or phase. Moreover, because these shifts were similar across the RGC population (Figure 1I), even the largest applied contrast changes resulted in only small distortions of the relative latencies between RGCs.

The latency code of fast-Off cells can be understood with a quantitative model of retinal circuitry [13], [16]. Fast-Off cells receive rectified excitation from both On- and Off-bipolar cells, which explains why they fire both on brightening and dimming of the receptive field. But the activation of On-bipolars is slower than for Off-bipolars, which explains why a brightening leads to spikes with longer latency. For gratings of different spatial phase, the receptive field experiences varying amounts of dimming and brightening, and thus the latency varies periodically with the spatial phase.

### A Neuronal Model that Computes with Spike Latencies

How can downstream visual circuits take advantage of this information encoded in the latency of ganglion cell spikes? Ideally, neurons in the recipient population should already perform a substantial computation, extracting visual features that are not represented by individual ganglion cells. With this goal in mind, we explored the capabilities of what is perhaps the simplest model of post-synaptic processing: a single integrate-and-fire neuron.

**Figure 2:**
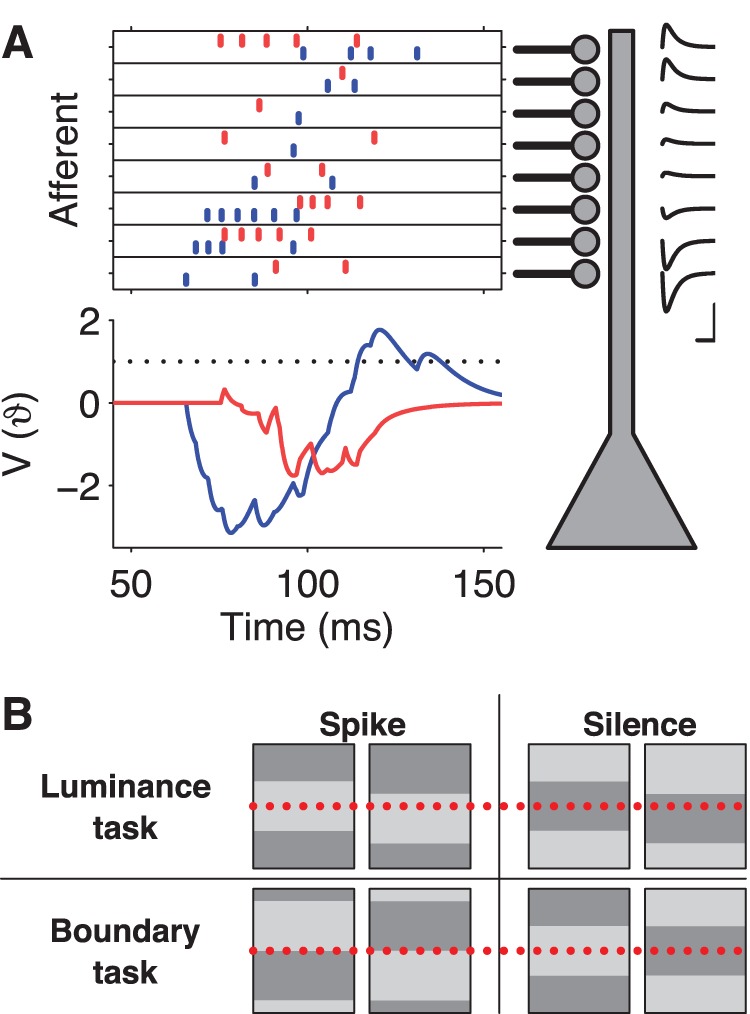
Spike-timing computations based on retinal spike trains. (**A**) Schematic of the modeled readout neuron. The neuron receives input from multiple afferents (top left). Each afferent spike produces a PSP of stereotyped shape with an amplitude that depends on the synaptic strength (insets top right, x-scale = 20 ms, y-scale = spike threshold). For some RGC spike patterns (e.g. the one in blue), excitatory and inhibitory input spikes arrive segregated in time such that the resulting PSPs sum to a peak voltage, 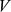, above the spike threshold (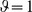), and the model neuron fires an action potential (bottom left, blue trace). For other patterns (e.g. red), excitation and inhibition interfere such that the peak voltage (red trace) remains below threshold and the model neuron remains silent. (**B**) Two visual categorization tasks based on the grating stimuli. Top: The luminance task asks whether a particular location in the field (dotted line) is bright (left) or dark (right). The readout neuron should fire in the former case but not in the latter; the opposite rule is another version of this task. Bottom: The boundary task asks whether a particular location (dotted line) has a boundary of either polarity (left) or no boundary (right).

The model neuron receives synaptic inputs from the population of RGCs (Figure 2A, top). These inputs may be of variable strength and either excitatory or inhibitory – the latter perhaps via a fast interneuron that introduces minimal delay [17], [18]. Neuronal processing occurs in episodes during which the afferents fire a volley of spikes (Figure 1A), as observed following visual saccades. Depending on the relative timing of these incoming spikes and their respective synaptic efficacies, the summed post-synaptic potentials from all synaptic inputs will either cross the neuron’s firing threshold or not (Figure 2A, bottom left). By either producing a spike or not, the model therefore classifies the input firing patterns into “target” (spike) and “null” (no spike) patterns. To accomplish a desired division of target and null patterns, one must adjust the synaptic strengths of the inputs appropriately. Recently, a synaptic learning rule has been introduced that finds the synaptic weights appropriate for a given classification task and operates successfully for a broad range of spike-time-based codes [14]. The integrate-and-fire neuron model, equipped with classification based on a single output spike and the associated learning rule, has been called the “tempotron” [14]. We will adopt this name as short-hand for the classifier model, even in cases where the appropriate synaptic weights are found by some other fitting procedure.

In the context of the above eight-grating experiment, we defined two image classification tasks, each requiring the detection of a specific visual feature. In each task, two of the eight gratings were defined as target stimuli to be discriminated from two other gratings that were null stimuli. In the first task, termed “luminance task”, the stimuli were grouped according to the luminance level at a certain location in the visual field (Figure 2B). This means that the tempotron had to discriminate a pair of neighboring gratings against their polarity-inverted complements, for example by discriminating gratings 1and 2 against 5 and 6 (Figure 1A). In the second task, the “boundary task”, stimuli were grouped by the presence or absence of a luminance boundary at a certain location, regardless of the sign of that boundary (Figure 2B). Specifically, one grating and its polarity-inverted complement had to be discriminated against another polarity-inverted pair, for example gratings 1 and 5 against 3 and 7 (Figure 1A). Intuitively, the luminance task is simple because it groups together stimuli that are very similar. The boundary task is harder because it groups stimuli that are as different as possible.

### The Tempotron can Classify Diverse Visual Features

**Figure 3:**
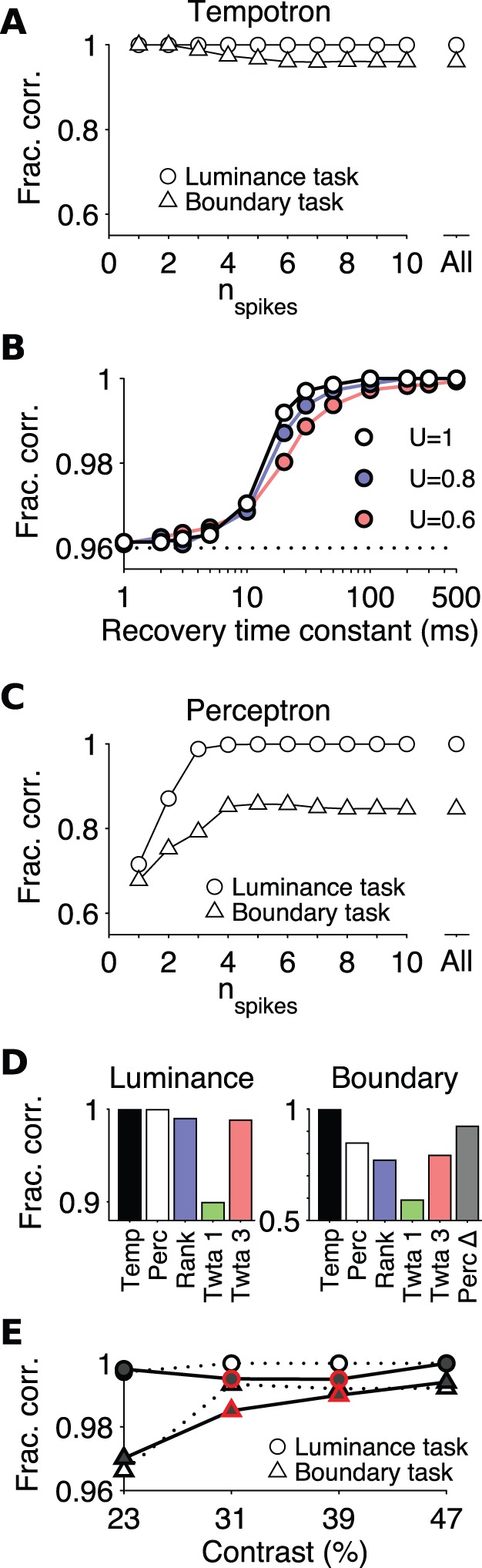
Performance of the tempotron and other decoders on RGC population responses. (**A**) Performance of the tempotron on the luminance (circles) and boundary (triangles) tasks in the highest contrast condition. Results are averaged over all realizations of each task and two separate populations of simultaneously recorded fast-Off RGCs, one with 7 neurons, the other with 8. The fraction of correct stimulus classifications is shown as a function of the maximal number of spikes admitted from each RGC. (**B**) Effect of synaptic depression on performance in the “all spikes” condition of the boundary task in (**A**). The fraction of correct responses is plotted as a function of the recovery time constant 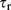 of synaptic depression and the synaptic utilization parameter 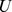, which determines the degree of depression (cf. Equation 5); 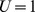 (maximal depression, black), 0.8 (blue), 0.6 (red). The dotted line indicates the performance with static synapses from (**A**). (**C**) As in (**A**), but performance of the perceptron decoder based on spike counts. (**D**) Comparison of the tempotron (Temp) and perceptron (Perc) peak performances from (**A**) and (**C**) with other timing-based readout schemes for the luminance (left) and boundary (right) tasks: Rank-order decoder (Rank), temporal-winner-take-all decoder using the first spike (Twta 1) or the first three spikes (Twta 3). The boundary task includes performance of a perceptron with an optimized integration window of 80 ms duration (Perc **Δ**). (**E**) Contrast dependence of tempotron performance in the luminance (circles) and boundary (triangles) tasks when using at most the first spike of each afferent ganglion cell. The fraction of correct classifications was measured separately within each of four contrast conditions (x-axis) on the basis of a seven-cell input population of RGCs. Open symbols with dotted lines: after training on all four contrast levels. Filled symbols with solid lines: after training only on the lowest and highest contrast levels. Note that the tempotron performs well even when generalizing to intermediate stimulus contrasts that were not encountered during training (colored symbols).

We provided the tempotron model with spike trains recorded simultaneously from a population of retinal ganglion cells and searched for the set of afferent connection strengths that solves each of the tasks specified above, using the tempotron learning rule. The tempotron’s readout performance was then measured by the fraction of trials on which the stimuli were classified correctly. Based on two separate populations of 7 and 8 simultaneously recorded RGCs, respectively, the tempotron learned both tasks very well (Figure 3A). The luminance task was accomplished without errors and the boundary task with a mean error of only ∼4%. We confirmed the generality of these results by, firstly, random resampling of the ganglion cell populations from our total pool of RGCs and, secondly, cross-validation on the basis of separate training and test sets (see Materials and Methods).

An advantage of a temporal code is that information may be available already with the arrival of the first spike and thus allow faster processing than codes that rely on counting spike numbers over extended time periods. To test the limits of rapid processing, we trained the tempotron using only the first spike or a subset of spikes from each afferent. Interestingly, discrimination on the boundary task improved with decreasing number of spikes, reaching error-free performance when only the first spike was admitted to the decoder (Figure 3A). Clearly, the timing information contained in the first spike from each RGC after a saccade is sufficient to perform these computations. Subsequent spikes in the burst interfere, though only slightly, with performance in the present task. This finding supports the idea that, for some visual tasks, processing could proceed as rapidly as possible by operating already with the timing of the very first spike. The solution of other visual tasks, on the other hand, may be accomplished by different readout neurons that rely on the subsequent spikes. For the RGC type considered here, for example, the number of spikes in the burst contains information about stimulus contrast [13].

How might a biological tempotron restrict its operations to the first spike of a burst on each afferent? A plausible mechanism is short-term synaptic depression, commonly observed in visual pathways [19], [20]. To explore this, we implemented a well-known model of synaptic dynamics [21] at each input to the tempotron. In this model, each action potential uses a fraction 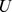 of synaptic resources for transmission – for example readily releasable vesicles – which then recovers with time constant 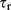. If 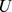 is sufficiently large, and 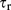 is long compared to the interspike intervals in a burst, synaptic depression will strongly discount all spikes but the first. As shown in Figure 3B, this synaptic depression can indeed enhance operation of the tempotron on the boundary task to near perfect performance, and this holds over a wide range of the dynamic parameters.

### The Tempotron Outperforms Other Readout Models on the Boundary Task

The “rate code” hypothesis stipulates that downstream visual areas extract image information from the firing rates of the ganglion cells. To evaluate the performance of the tempotron, it is therefore interesting whether a neuronal decoder could achieve similar discriminations by using only the spike count of bursts from ganglion cells and not their timing. Thus, we implemented a second readout neuron that follows the classic perceptron model of neural integration [22], [23]. Analogously to the tempotron, this model neuron also receives ganglion cell inputs from its afferent sources and adjusts their scalar synaptic efficacies through an iterative learning rule. However, unlike the tempotron’s integration of incoming spike trains in continuous time, the perceptron evaluates each afferent’s spike count within a fixed input window of 150 ms after stimulus onset, and its classification decision is given by thresholding the weighted sum of the incoming spike counts.

The perceptron performed the luminance task very well (Figure 3C): Clearly the RGC population contained enough neurons whose firing rates encoded whether the receptive field is dark or light. However, the perceptron performed poorly on the boundary task, with a mean error rate of ∼15% (Figure 3C). Unlike the tempotron, the perceptron’s performance degraded as the number of admitted spikes was reduced (Figure 3C). When limited to just the first spike from each RGC, not surprisingly, the perceptron failed at both tasks; its meager residual performance was owed to ganglion cells that failed to fire at all for some stimuli. A somewhat better performance was obtained by limiting the perceptron spike count to a fixed time window after stimulus onset, but this window length required optimization for each task (80 ms for the boundary task, Figure 3D). Furthermore this readout scheme requires that the decoder know the absolute time of the saccade, whereas the tempotron operates only on relative spike times. Note that tempotron and perceptron models had the same free parameters, namely the synaptic strengths. Yet the tempotron operating with spike times was superior to the perceptron operating with spike rates: It could perform a more complex visual task, and it solved the computation with fewer spikes, hence also in much shorter time.

Another useful benchmark for comparison are models of neural processing that operate on individual spikes, but only consider the temporal order of spike arrival on different afferents, not their spike times. In one of these readout schemes, the “temporal winner-take-all model”, each afferent votes for one of the two outcomes of the visual computation, and the afferent that fires first determines the decision [24]. This model performed moderately on the luminance task, but failed entirely on the boundary task (Figure 3D). In a more general version of this readout scheme [25], we considered the first three spikes in the afferent population and computed the outcome by majority vote of those neurons. This model solved the luminance task well, but was still worse than the perceptron on the boundary task (Figure 3D). Considering more than three spikes did not improve the model’s performance on either of the tasks.

While the temporal winner-take-all model bases the decision entirely on one or a few neurons that fire first, an alternative “rank order decoding” model is sensitive to the temporal order of the first spikes from all its afferents [26]. Like the tempotron, this model has the synaptic weight of each afferent as a free parameter and in addition a factor that accounts for progressive desensitization of the recipient neuron. The rank order decoding model also solved the luminance task well, but again failed on the boundary task (Figure 3D). These alternate models were designed for rapid neural computation by operating on the first few spikes in a sensory episode. For each of these models, we assumed that the appropriate set of spikes could be selected, such as each afferent’s first spike or the first three spikes of the population, without regard to the mechanisms that might accomplish this. Nevertheless, these models could not perform a complex classification like the boundary task in a single synaptic stage.

### The Tempotron’s Performance is Contrast-invariant

A hallmark of sophisticated receptive fields is that they are highly tuned for certain visual features while remaining non-selective for other features. For instance, neurons in the face area in primate cortex respond selectively to a specific face, independent of the retinal position of that face [27]. The decoder of the boundary task already has this character: It is selective for the spatial location of a light-dark edge independent of its polarity. We further explored the invariance of the tempotron to changes in another dimension of the stimulus, namely its contrast. In these experiments, each of the eight gratings was presented at different contrast levels, randomly interleaved. The model neuron was again trained to classify stimuli in the luminance and boundary tasks, but this time invariantly with respect to four different levels of stimulus contrast, ranging from 23% to 47%. This contrast range provided a substantial increase in the drive to the ganglion cells, with the average spike number for the preferred phase growing by 60% from 3.67 to 5.61. Below this range an increasing fraction of ganglion cells failed to respond to all used stimulus phases.

We found that the tempotron adjusted easily to this added requirement, with essentially zero errors on the luminance task, and on average 1.2% errors on the boundary task (Figure 3E). Moreover, the tempotron was able to generalize effectively to new contrast values that it never experienced during the training phase (Figure 3E).These observations suggest that the tempotron makes use of patterns in the ganglion cell responses that remain invariant under changes of stimulus contrast. Indeed, an increase in stimulus contrast led to shorter absolute latencies by up to several tens of milliseconds (Figure 1I) [13]. However, this effect is of similar magnitude for different cells in the population and across stimuli, so that the relative latencies between RGCs vary rather little (Figure 1I). The tempotron has no access to absolute time or stimulus onset; instead, it operates only on relative latencies, and thus its performance remains largely contrast-invariant.

### Mechanisms of Tempotron Computing

How does the tempotron accomplish these tasks? It helps to inspect the simplest version of the readout that uses just two RGCs for input, using just the first spike each, corresponding to strongly depressing synaptic transmission. This reduced scenario is amenable to an analytical treatment for finding the optimal synaptic weights and thereby to a conceptual characterization of the types of solutions as seen below. We analyzed the circuit’s classification performance for all 89 available pairs of simultaneously recorded RGCs, and found that among these ∼89% could solve the luminance task and ∼24% could solve the boundary task with error rates below 5%.

**Figure 4:**
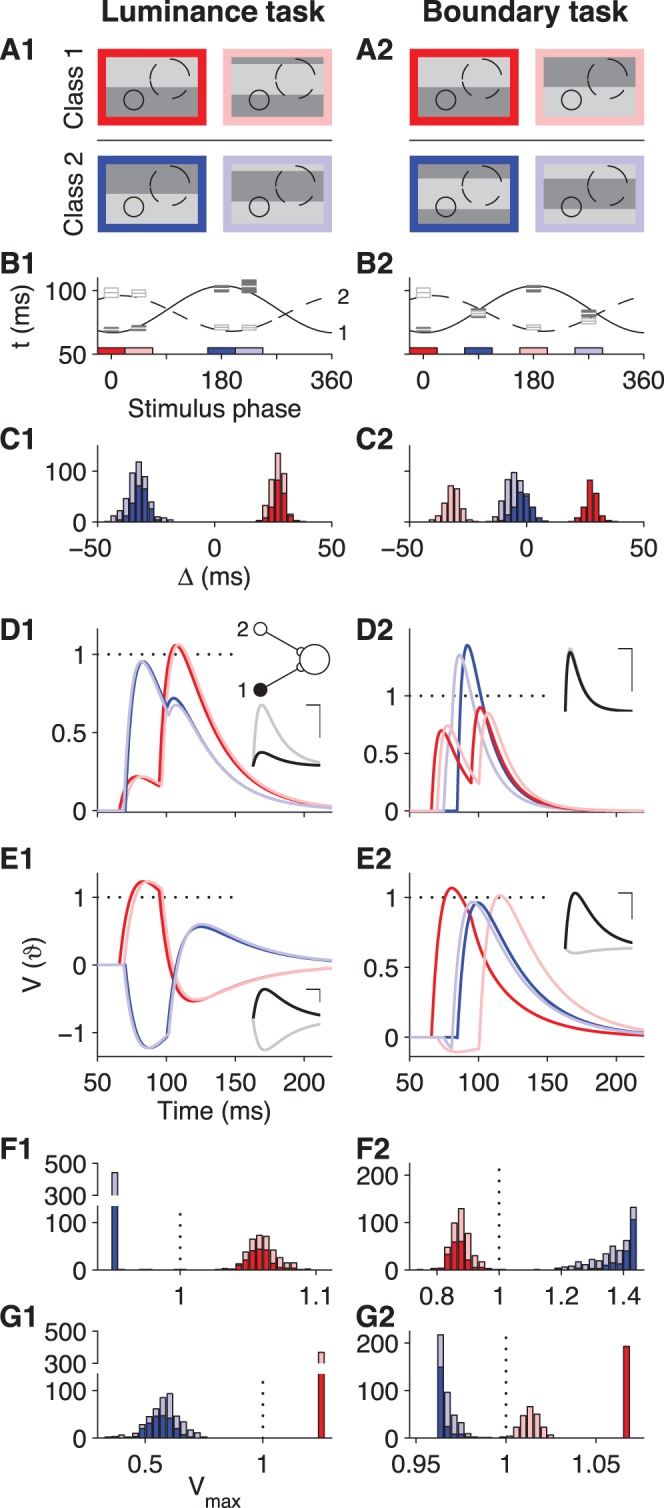
Mechanisms of spike-latency-based neuronal computing. Illustration of sample tempotrons that solve the luminance (left) or the boundary (right) task. Each tempotron receives inputs from only two RGCs, and only their first spikes are processed. (**A**) The four grating stimuli that define each task and the relative locations of the two recorded RGC receptive fields. Solid line: Cell 1; dashed line: Cell 2. (**B**) Latency tuning of the two RGCs under the eight gratings. Lines show fitted cosine tuning curves. Colored horizontal bars highlight the spatial phases of the four stimuli used in each task, color-coded as in (**A**). For each of these stimuli, boxes depict the mean ±1 SD of the measured first-spike latency. (**C**) Histograms of the differential latencies 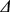 of the two RGCs during the four task-relevant stimuli, color-coded as in (**A**). (**D**) Sample voltage traces of a tempotron that solves the task, color-coded according to the four stimuli. The input spike times of this example represent the median differential latencies observed experimentally. Horizontal dotted line depicts the spike threshold. Inset: The minimal readout circuit with two RGCs connected to one postsynaptic neuron. Traces show the PSPs of cell 1 (black) and cell 2 (gray) that underlie the solution shown in the main panel. Scale bars depict 20 ms in the x-direction and half of the spike threshold in the y-direction. In these implementations, both afferents are excitatory. **D1**: 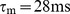, **D2**: 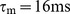. (**E**) Like (**D**), but in these solutions, one afferent is excitatory and the other inhibitory. **E1**: 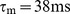, **E2**: 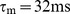. Note that the solution in (**E2**) encodes the identity of the stimulus within the target class by the latency of the output spike: While early responses signal the dark red stimulus, later responses signal the light red stimulus. The membrane time constants in (**D**) and (**E**) were chosen to minimize the generalization errors for the sample distributions of (**C**) in the investigation of threshold noise (Materials and Methods). (**F**) Histogram of the peak voltage of the tempotron in (**D**) for all experimental trials, color-coded by stimulus as in (**A**). Note that the target and null stimuli are well separated on either side of the threshold (dotted vertical line). (**G**) Like (**F**), but for the solutions in (**E**).

To understand how this is achieved, consider two RGCs whose receptive fields are separated by about one grating bar, or by a phase of 180° (Figure 4A). Under the luminance task, the two RGCs experience opposite light intensities and thus fire at different times (Figures 4A1 and B1). For one stimulus class, cell 1 fires first, and for the other class, cell 2 fires first (Figure 4C1). Hence, the tempotron must merely determine the order of firing among these two afferents. This can be accomplished in two ways. One solution uses positive synaptic weights of unequal magnitude (Figure 4D1). Each afferent’s PSP by itself remains below threshold. If the stronger one fires first, its effect decays by the time the weaker PSP arrives, and the sum fails to cross threshold. By contrast, if the weaker one fires first, this gives enough of a boost to the second stronger PSP to cross threshold. Another solution combines excitation and inhibition such that, again, the threshold is crossed only in one order of firing, and not the opposite (Figure 4E1). Both solutions map the two stimulus classes into clearly distinct values of the model’s peak membrane voltage (Figures 4F1 and G1).

The boundary task (Figure 4A2) presents a more intricate challenge. One stimulus class leads the two RGCs to fire almost synchronously (Figure 4B2) because both receptive fields are straddled by a luminance boundary and thus experience the same input. The other class makes them fire at different latencies, but neuron 1 leads under one stimulus and neuron 2 under the other (Figure 4B2). The histogram of relative latencies now has three peaks (Figure 4C2), and the readout neuron must separate the events in the central region, when the two RGCs fire in near synchrony, from the regions on either side. To solve this task, both afferents are assigned the same positive weight. If the two spikes occur simultaneously, their PSPs superpose and cross threshold. If one or the other fires earlier, its effect decays, such that the summed PSP remains below threshold (Figures 4D2 and F2). Again, another solution can be found that combines excitation and inhibition, this time with reversed roles of the target and null classes (Figures 4E2 and G2). In this case, synchronous firing of the RGCs leads to destructive interference of the excitatory and inhibitory inputs. By contrast, temporally separated firing allows the supra-threshold excitatory afferent to trigger a post-synaptic spike. See also Figure 2A for an implementation of this solution using a larger population.

**Figure 5:**
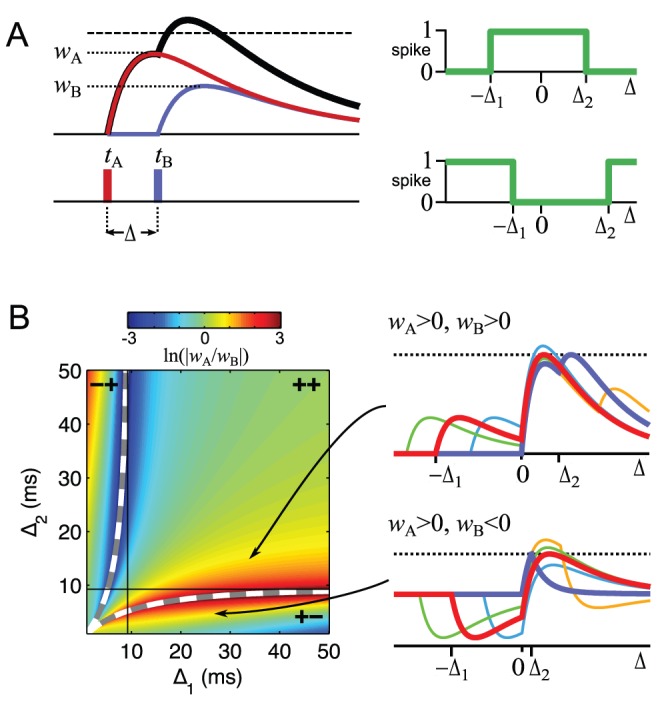
Synaptic solution space of a ganglion cell pair decoder. (**A**) In this simple version of the decoder, each of the two afferents fires exactly one spike per stimulus presentation at times 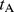 and 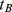. The two spikes produce PSPs with identical kinetics but different amplitudes 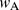 and 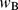. The relative latency 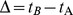 of the two spikes determines whether the combined PSP crosses threshold for a spike. This classifier divides the range of 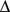 into three regions: 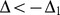, 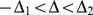, and 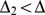. Depending on the two synaptic weights, the decoder fires only in the middle region (top right, e.g. Figure 4D2) or only outside that region (bottom right, e.g. Figure 4E2). (**B**) One can prove that for any desired location of the boundaries 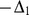 and 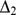 there is a combination of synaptic weights 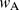 and 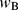 that provides the correct classification. Here this solution space is computed using PSP kinetics with 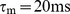 and 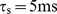. The left hand plot shows for any combination of 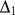 and 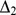 the ratio of synaptic weights 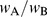 that solves the task. In the region marked “++” both synaptic weights are positive and the readout neuron fires inside the range 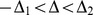. In the regions marked “+–” and “–+” the weights are of opposite sign, and the readout neuron fires outside the specified range. Dashed lines indicate the boundary between the three types of solutions. Their location depends on the PSP kinetics and asymptotically approaches the time-to-peak of the PSP (solid lines). See Materials and Methods for details. The right hand plots illustrate two specific solutions for the 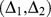 combinations indicated by the arrows. In each case, the PSP is shown for several different latencies 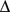; bold lines correspond to the limiting cases 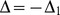 and 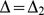, for which the PSP just reaches threshold.

The tempotron’s ability to separate the central region of the relative latency histogram from the two adjoining ones underlies its solution of the boundary task. One can show analytically that the tempotron with two inputs can always accomplish a tripartite dissection of the range of relative latencies around zero, no matter where the two desired decision boundaries lie (Figure 5, see also Materials and Methods). Furthermore, there is a broad range of decision boundaries for which purely excitatory solutions are available (“++” in Figure 5B). Finally, if one allows shorter or longer PSP time courses, then every possible tripartite dissection can be served by a purely excitatory solution as well as a mixed excitation-inhibition solution, underscoring the versatility of this simple readout architecture. Although this analysis is specific to two inputs with one spike each, it provides insight into the ability of the tempotron to use timing information to perform rather complex computations in a single stage. As seen earlier, additional spikes may interfere somewhat with performance, but should not affect the fundamental solutions of tempotron operation – and their effect on the readout may be minimized by synaptic depression.

### Noise Robustness and Speed of Tempotron Computing

**Figure 6:**
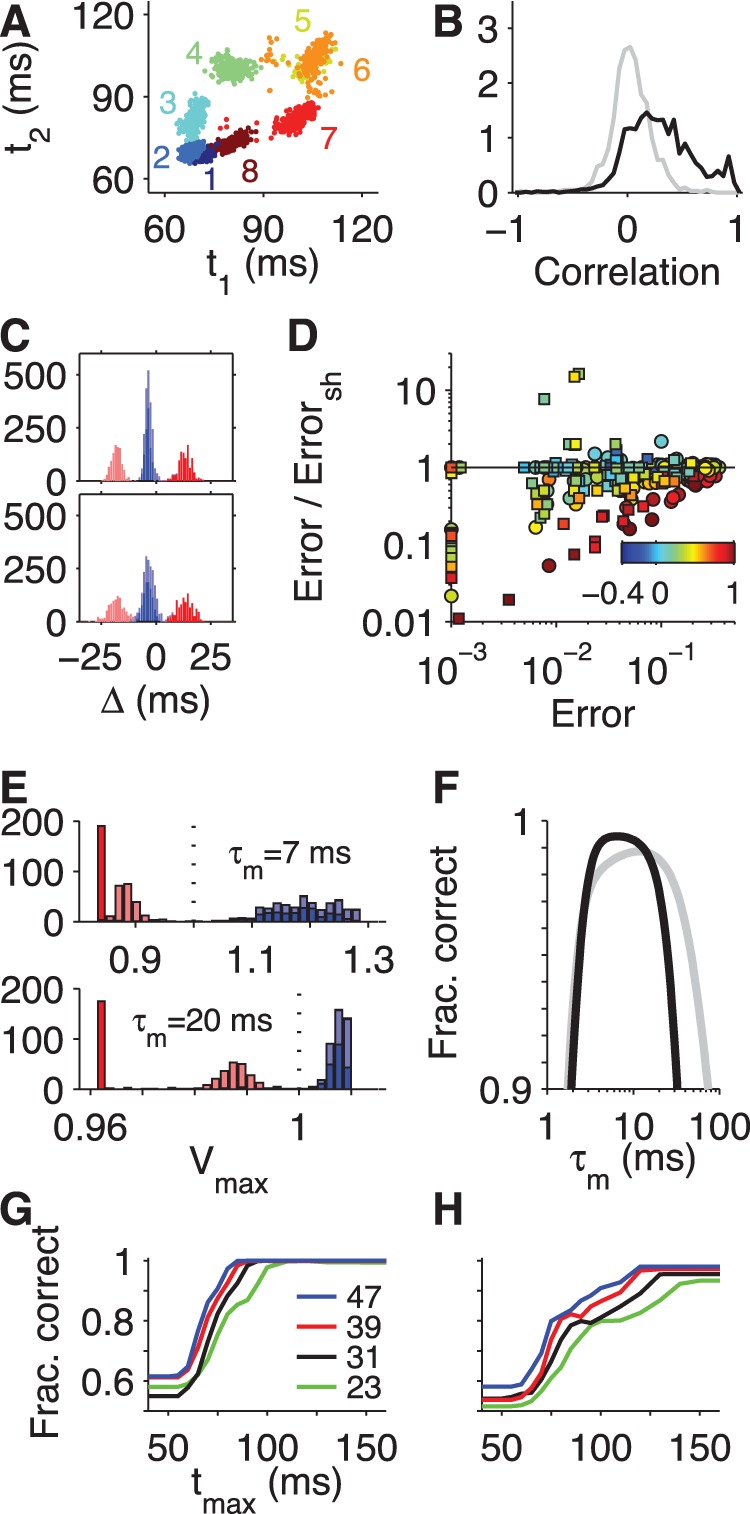
Effects of spike-time noise, threshold noise, and readout time on tempotron performance. (**A**) Scatterplot of first-spike latencies of two RGCs on multiple trials for each of the eight stimulus phases in the highest contrast condition. For this cell pair, the latencies covary, with an average correlation coefficient of 0.46 over all eight grating phases. (**B**) Histogram of correlation coefficients for first-spike latencies observed for all simultaneously recorded cell pairs, stimuli, and contrasts (black). Note the excess of positive correlations. As a control, the gray line shows the analogous histogram obtained when correlating latencies of the two cells separated by one stimulus trial. (**C**) Top, histogram of relative latencies for the cell pair of (**A**) for the boundary task of Figure 4C2. Bottom, the same histogram of relative latencies, but obtained from shifted trials. Note the increased dispersion of the relative latencies and the increased overlap between the red and blue peaks. (**D**) Effect of latency correlations on readout performance. For all simultaneously recorded cell pairs, we obtained the minimal tempotron error rates with inputs from simultaneous trials and with inputs from shifted trials. The ratio of these two error rates is plotted against the error rate obtained for simultaneous trials. Squares: luminance task; circles: boundary task. Symbol colors represent the mean latency correlation across the four stimuli that constitute a given task (color bar). Note that most of the points lie below unity, showing that in most cases the readout performance degrades when trials are shifted and latency correlations destroyed. (**E**) Distributions of the peak voltage of the tempotron for the boundary task, based on the distributions of relative latencies shown in (**C**) (top; same color code). The two voltage distributions result from optimizing the tempotron weights for different PSP kinetics, namely 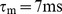
(top) and 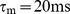 (bottom). In this example the shorter PSPs generate a much larger separation between the maximal voltages for target and null stimuli (red vs blue), such that the spike readout would be more robust to any noise in the neuron’s threshold. (**F**) Optimal classification performance on the boundary task with the input cell pair of (**C**) and assuming a Gaussian threshold noise whose standard deviation is 5% of the mean synaptic weight magnitude. The error was minimized for each PSP time constant (x-axis) over the synaptic efficacies for the purely excitatory solution (black, Figure 4D2) and the mixed solution with one excitatory and inhibitory input (gray, Figure 4E2). (**G**) Performance of optimal tempotrons operating with a pair of RGCs on the luminance task as a function of the maximal allowed latency, 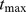, of input spikes. The fraction of correct classifications was averaged over all input cell pairs that allowed for error-free performance in the highest contrast condition at large 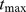. Results are plotted for different values of the stimulus contrast (indicated in %). (**H**) As in (**G**) but for the boundary task and the fraction of correct classifications averaged over all input cell pairs with errors below 5% in the highest contrast condition at large 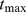.

With this understanding of the basic computation implemented by the tempotron readout neuron, we now consider some limitations to its performance. One obvious limitation is imposed by the spike-timing precision of the afferent neurons. Large noise in spike timing will lead to broad peaks in the relative latency histogram (Figure 4C). If the peaks from different stimulus classes overlap, a readout neuron has no chance of separating them. For the recorded retinal ganglion cell spike trains, the jitter of absolute latencies was on the order of a few milliseconds [13]. The effects of this noise are diminished, however, by the fact that the trial-to-trial variations in latency are correlated across ganglion cells (Figures 6A–C) [13]. Spike times of different neurons tend to shift back and forth together, possibly because of small gain changes in the circuit that depend on the common stimulus history or because of shared input noise [28]. Therefore the jitter in relative latency is considerably smaller than one might assume if the two RGCs had been recorded independently. We found that this makes a substantial difference to neuronal classification performance (Figure 6D); strongly correlated cell pairs had considerably smaller error rates when the actual simultaneously recorded data were analyzed as compared to the scenario where their correlations were broken up by shuffling the trials. This shows that spike time correlations increase robustness to spike time jitter, and it emphasizes the importance of simultaneous population recordings when one considers computations involving spike timing.

Even if the input spikes are timed reliably, a realistic detector neuron will experience some noise unrelated to the inputs, so that effectively its threshold varies from trial to trial. If the maximum voltages produced by null and target stimuli are well separated, the model is robust to such threshold noise, but otherwise it will experience classification errors (Figures 6E and F). The distribution of maximum voltages in turn depends on the temporal window of the postsynaptic potential (Figure 6E). We analyzed this sensitivity to threshold noise in the simple case of just two inputs, each of which fires one spike. For the difficult boundary task, we found that the optimal postsynaptic integration time when processing RGC responses was on the order of milliseconds to a few tens of milliseconds (Figure 6F). As expected this time scale is comparable to the latency differences that need to be discriminated, tens of milliseconds (Figure 1B), but clearly a rather broad range of PSP time constants will work. In this regime, the tempotron accomplished near perfect performance even if the threshold was corrupted by noise equal to 5% of the PSP amplitude (Figure 6F, see also Materials and Methods). Thus the temporal computations are robust as long as the time scale of postsynaptic integration is chosen appropriately.

For the purpose of rapid neuronal processing, the speed of the tempotron’s computation is of central importance. We explored this further by restricting the tempotron’s input spikes to those arriving within a certain limited time window after stimulus onset. As this window is extended, performance rises from chance to perfection (Figures 6G and H). Note that at larger stimulus contrast, less time is required to perform the classification tasks; this follows directly because all absolute response latencies are shorter at high contrast (Figure 1I) [13]. Interestingly the boundary task always requires more time than the luminance task. Referring to the histogram of relative latencies (Figure 4C), one sees that the luminance task can be decided as soon as the short-latency spikes have arrived (Figure 6G), whereas the boundary task requires the neuron to wait through the period of intermediate latencies (Figure 6H). In general therefore, one expects that the timing of the tempotron response will vary with the nature of the task. The timing of the output spike can also carry further information about the input stimulus even within the target class (e.g., Figure 4E2), and this may be used by spike-timing computations at the next stage of neuronal processing.

### The Tempotron can Implement Orientation Selectivity with and without Phase Invariance

The observation that the tempotron allows detection of boundaries independent of polarity led us to explore the detection of other visual features. A very common task used in human psychophysics and animal experiments is the discrimination of grating displays oriented at different angles. In most of these studies the grating is presented at random phase, due to uncontrolled eye movements, so the visual computation requires detecting the grating orientation independently of its phase. In the mammalian visual cortex one finds neurons that may contribute to this task: so-called “simple” cells are selective for gratings of a particular orientation and phase, whereas “complex” cells are selective for an orientation, but invariant with phase [29]–[31]. Can a tempotron perform this task based on the raw spike trains from retinal ganglion cells?

**Figure 7:**
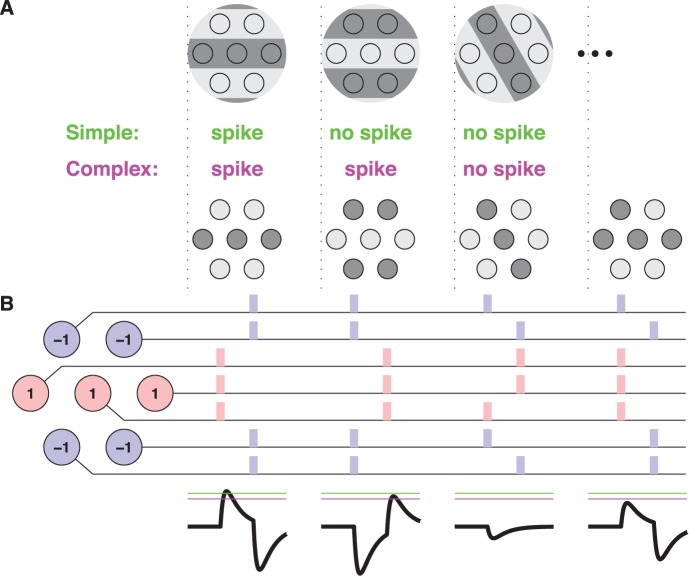
Schematic model of orientation selectivity by latency processing. (**A**) In this model, a population of 7 RGCs is stimulated by a sudden appearance of a bright/dark grating, and the resulting spike trains are processed by a tempotron. To emulate a cell that detects a single horizontally oriented pattern, reminiscent of a cortical simple cell, the tempotron should fire to the preferred grating (left), but remain silent to its inverse (middle), a rotated version (right), or any other pattern of illumination. To detect a horizontal grating independent of polarity, the tempotron should fire both to the preferred grating (left) and its inverse (middle), but reject all other patterns. (**B**) A set of synaptic weights assigned to the 7 RGCs (left) that solves this problem. Each RGC fires a spike either early or late (if its receptive field turns dark or bright, respectively) with a relative time difference of 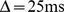 (Figure 1H). The resulting spike patterns produced by 4 different stimuli (top) are shown, with colors indicating each spike’s excitatory or inhibitory contribution. Bottom panels show the postsynaptic voltage traces elicited in the tempotron (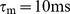). All 126 binary stimulus patterns other than the preferred grating and its inverse produce a peak voltage of 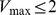 in units of the unitary PSP amplitudes. The preferred grating and its inverse produce the two highest values with 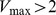; in the present model, this occurs if 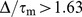. Because of the order of excitation and inhibition, the preferred grating always elicits a higher peak voltage than the inverse grating. Hence, if the spike threshold 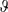 is high (green line) the tempotron detects a single pattern, if 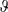 is lower (pink line) it detects horizontal gratings of both polarities.

For illustration, imagine an array of retinal ganglion cells on a hexagonal lattice (Figure 7A). We consider stimuli that switch from uniform gray to an arbitrary pattern of dark and bright regions. Each RGC responds with a spike whose latency depends on the stimulus: short latency if the receptive field turned dark, longer latency if it turned bright. The tempotron receives inputs from a patch of seven such ganglion cells. For stimulus selectivity analogous to a simple cell, this neuron should fire for a horizontal grating with central dark bar, but remain silent if the same grating is rotated or inverted in phase (Figure 7A). Indeed we shall require the neuron to remain silent for all 127 bright/dark stimulus patterns other than the preferred grating. For complex-cell-like behavior, the postsynaptic cell should fire for the horizontal grating as well as its phase-inverted version, but remain silent for the other 126 stimuli. Although this may at first seem challenging, these stimulus selectivities are in fact achieved with a very simple pattern of synaptic weights (Figure 7B). Furthermore, the same synaptic weights will produce either simple or complex selectivity: A lowering of the firing threshold or a shorter PSP duration elicits a switch from selectivity for an individual pattern to phase invariance (Figure 7B). Thus the degree of invariance in the tempotron’s response could be controlled by modulating its effective integration time, for example depending on the amount of shunting conductance [32], [33].

**Figure 8:**
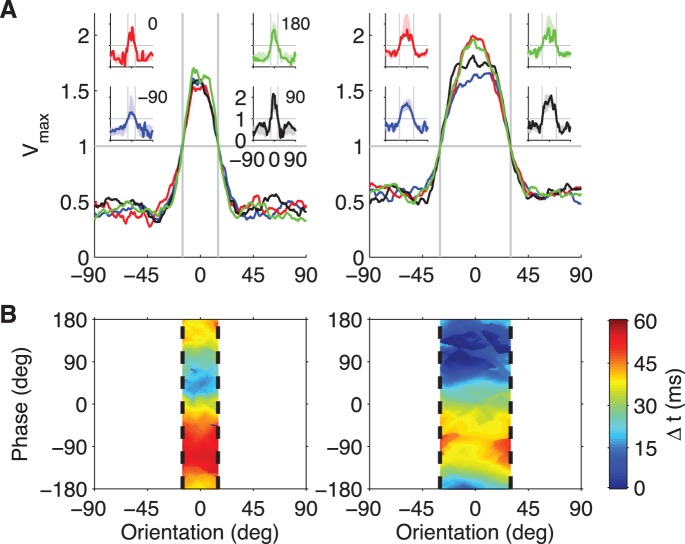
A tempotron model for orientation tuning with phase invariance. (**A**) Orientation tuning curves of a tempotron model designed to show phase invariance. Extending the schematic model shown in Figure 7, this tempotron received inputs from 200 model RGCs whose Gaussian receptive fields were randomly placed in the circular region over which the grating was presented. The latency of each RGC was cosine-tuned to the fraction of its receptive field covered by dark grating bars as in the experimental recordings (Figure 1). The tempotron was trained to fire in response to orientations within ±15° (left) or ±30° (right) independently of the grating phase. The curves show average orientation tuning curves of the maximal voltage for four different grating phases (–90°: blue, 0: red, 90°: black, and 180°: green). Averages were obtained over 14 (left) and 15 (right) independent RGC populations and tempotrons. For each tuning width, insets show the four orientation tuning curves of one individual tempotron, overlaid on the mean ±1 standard deviation of the populations (shaded areas). (**B**) Spike timing of the two tempotron models shown in the insets of (**A**) with narrow (left) and wide (right) orientation tuning. The latency of the output spike (color code) was measured relative to the shortest-latency spike of the tempotron and plotted as a function of orientation and phase of the stimulus grating. Each tempotron spiked only for orientations within the dashed vertical lines.

While this schematic example gives some intuition how orientation tuning with single spikes might work, it is restricted to a small number of stimuli and leaves open whether this can be accomplished using realistic retinal spike trains as input. To explore this, we set the goal of producing a model cell that responds to grating stimuli of arbitrary orientation and phase by firing reliably within a narrow orientation range, but entirely independent of the phase of the grating (Figure 8A). As inputs we used 200 model ganglion cells with randomly scattered receptive fields and response properties drawn from the experimentally observed population of fast-Off RGCs (Figure 1). The efficacies of their synapses onto the model readout neuron were trained by the tempotron rule, with the objective of obtaining a spike from the tempotron model if and only if the grating orientation is in a specified range, with a width of either 30 or 60 degrees.

We found that the trained readout neuron achieved precise orientation tuning in the specified ranges, regardless of the phase of the grating (Figure 8A). Interestingly, whereas the presence of the output spike depended only on the orientation of the stimulus grating, the exact time of the spike was strongly dependent on the phase of the grating (Figure 8B). We can only speculate whether there exist neurons downstream from the retina that attain orientation selectivity in this way, yet this analysis shows that the sophisticated receptive fields encountered in many cortical neurons can, in principle, be realized by computations with single spikes. It seems likely that a dedicated brain pathway for rapid image analysis would benefit from neurons that achieve orientation-selective and phase-invariant responses in a single stage of synaptic integration in order to facilitate rapid complex visual recognition processes.

## Discussion

This study was triggered by the observation that certain types of retinal ganglion cells implement an explicit spike latency code, in which the timing of a spike at the onset of image fixation encodes the spatial layout of the stimulus (Figure 1). We explored how downstream neurons of the visual system might compute with this code to extract features not represented by individual RGCs. It emerged that the simplest picture of a receiver neuron, the well-known integrate-and-fire model, already offers substantial capabilities for computation based on spike times. Using just a single spike per afferent fiber, this “tempotron” can perform basic visual tasks representative of biological feature detectors in the visual system (Figures 3, 7 and 8), while models that operate on the spike count or purely on the temporal order of afferent spikes fail on the more challenging tasks (Figure 3). The tempotron’s performance was highest when focusing on the first spike of each afferent (Figure 3A) and synaptic depression provides a plausible mechanism for this restriction (Figure 3B). With different sets of synaptic weights, the tempotron can implement qualitatively very different computations (Figures 4 and 7), while its output is invariant to stimulus contrast and robust to certain forms of noise originating in the retina (Figure 6). Finally, we found that the tempotron can achieve in a single synaptic stage orientation selectivity and phase invariance, a computation reminiscent of cortical complex cells, whereas conventional models of neural processing require multiple synaptic stages for this feat (Figures 7 and 8). The speed and versatility of tempotron computation recommends this mechanism for a rapid image-processing channel.

### Spike-time Computations

The notion that stimulus information can be extracted from the spike times of sensory neurons has been explored extensively [34], [35]. Indeed, the communication from retina to cortex by first-spike latencies has been modeled before [36], [37], and a hierarchical network of latency-decoding neurons has been shown capable of high-level visual tasks like face recognition [26]. In general, these arguments assume that sensory encoding occurs in discrete episodes, such as visual fixations, olfactory sniffs, or somatosensory whisking, and that, for each sensory neuron, stronger stimuli produce spikes earlier in the episode. In this way, the temporal order of firing encodes the stimulus, allowing decoding in downstream regions by reading the firing sequence. To this framework, we add two important concepts: more elaborate and realistic sensory encoding and a concrete proposal for a simple but powerful biophysical decoding mechanism that extracts information from multi-neuronal spike latency patterns.

First, the retinal ganglion cell signals in our study were actually observed experimentally, and we focused on a cell type with specialized response properties. These neurons fire bursts of spikes in response to almost any stimulus, and the onset time of the burst depends on the proportion of light and dark regions in the receptive field [13]. Second, we offer a concrete mechanism to exploit this spike latency code in downstream brain regions in a way that goes beyond a mere readout of the stimulus and already begins certain computations. The course of the computation is embodied entirely in the synaptic strengths of the afferents, which we obtained via the tempotron learning rule [14] or by exhaustive search. In nature, the synaptic strengths may well be hard-wired or learned by some activity-dependent mechanism. We do not consider this question further here, except to note that there exists a biologically plausible synaptic learning rule that approximates the tempotron rule [14] and that other mechanisms for learning specific spike patterns have been explored [38].

At the heart of tempotron computing lies its sensitivity to the temporal relation between inputs from different afferents [14]. For example, solving the boundary task requires an assessment whether two stimulus components are the same or different. The tempotron can determine easily whether two neurons fire at different times, regardless of their order (Figures 4 and 5). This is equivalent to solving the so-called “XOR problem”, a task that is notoriously impossible for a perceptron model, which operates by linear summation of a scalar response measure over its inputs [22], [39]. With just two inputs, a tempotron can partition the stimulus space into three separate regions (Figures 4 and 5), whereas the perceptron can only cut the stimulus space in two. Similarly, the other models that rely on the rank order of spikes can distinguish different orders of arrival among the afferent spikes, but they cannot separate coincident from non-coincident patterns. This explains why the tempotron performed the boundary task better than the perceptron or the other considered models. The same principle is behind the tempotron’s ability to detect the orientation of a grating regardless of its phase in a single synaptic stage (Figures 7 and 8).

### Biological Implementation of Spike-time Computing

Given the potential power of tempotron processing, one wonders whether nature actually exploits computation with spike latencies. In the following, we speculate about the possibility of such pathways in the mammalian visual system and discuss the necessary ingredients for implementing them. Beginning already in the retina, visual information is processed in many parallel pathways, each presumably playing some unique role for the animal’s overall visual performance [40]. Spike-time computing would be particularly useful in a processing pathway where speed is essential, for example for a rapid coarse assessment of the new visual scene after a saccade. Indeed, humans and monkeys can make high-level decisions about images already 100–200 ms after light strikes the retina [41]–[43], and cortical neuronal signals have been measured that support such rapid classification both in monkeys [44] and in humans [45]. This suggests that some aspects of visual processing occur at high speed. Presumably other parallel pathways through the visual brain operate with more leisure – for example for a detailed inspection of the scene throughout a visual fixation – and these may well use a different neural code. Thus we suggest that tempotron computing may be a hallmark of specialized pathways through the visual system, which benefit from the appropriate synaptic and cellular physiology.

#### Input from the retina

The On-Off retinal ganglion cells we considered are a special cell type that has been characterized extensively in the salamander retina [46], [47]. Mammalian retinas also contain multiple RGC types with On-Off responses [48]–[50]. On the other hand, On-Off ganglion cells are not essential for the proposed computations. The tempotron may equally well combine inputs from On cells and Off cells with overlapping receptive fields. The essential requirement for the present scheme is that On and Off responses follow different dynamics. For example, On and Off parasol cells of macaques differ in response latency by 10–15 ms [51], which could support spike-time computing at the onset of fixation.

#### Transmission through the thalamus

Relay cells in the lateral geniculate nucleus generally mirror the response properties of retinal ganglion cells. Often they are dominated by input from a single RGC and fire time-locked to the input spikes [52], [53]. There is little temporal dispersion of action potentials from the retina all the way to the visual cortex [54]. Thus the spikes arriving at the visual cortex maintain the essential timing relationships [55], [56]. Moreover, relative timing between pairs of thalamic neurons has recently been shown to encode the orientation of a moving grating [57].

#### Transmission to visual cortex

Each recipient cell in the cortex is within reach of ∼100 afferents from the thalamus [58], so this is clearly a site of spatial computations. A pathway that performs spike-time computing of the type considered above should meet certain conditions. First, the cortical neuron should receive strong afferents such that only a few, well timed PSPs are sufficient to reach the firing threshold. Indeed, the synapses from geniculate afferents are remarkably strong, and just a few spikes are sufficient to make cortical neurons fire [59]. Second, the integration time of postsynaptic neurons should be matched to the temporal structure of retinal activity. Based on the responses of salamander RGCs, we predicted an optimal integration time of ∼10 ms (Figure 6F), and this should be somewhat shorter in a mammal with faster responses. Indeed time constants of 2–9 ms have been measured in cat visual cortex [60], and a direct measurement of spike interactions from thalamic afferents revealed an integration time constant of 2.5 ms [61]. Thus it appears that both the strength of afferent synapses and the dynamics of postsynaptic integration are conducive to spike-time computing in cortical cells.

#### Intracortical circuits

The canonical view of cortical neural coding is that the information about relevant visual features is distributed among many cells, that individual neurons are noisy, that their synapses are weak but numerous, and that individual spikes have a negligible effect on connected neurons. While this picture seems less conducive to cellular computation based on afferent spike times, it is fair to say that the available experimental evidence leaves ample room for dedicated pathways within the cortex that operate differently [62]. In fact, the typical cortical neuron is rather silent, with maintained firing of 1 Hz or less [63]; on that low background activity, even a single spike triggered by a saccade can stand out effectively. Furthermore, within the sea of weak synapses, one finds a conspicuous subset of very strong connections, where individual spikes evoke postsynaptic potentials of several millivolts [64], [65]. These circuits could provide an effective substrate for tempotron computation.

#### The role of inhibition

Although the tempotron can solve visual tasks with excitation only (Figures 4 and 5), the most versatile application of the model assumes that each afferent could, in principle, contribute net excitatory or inhibitory signals. Indeed, this kind of synaptic circuitry is available, at least in the early sensory pathways. At the retino-thalamic synapse, an individual spike from a retinal ganglion cell can evoke both excitatory and inhibitory postsynaptic currents in the projection neuron [17]. Although the inhibition arrives via an additional interneuron, its delay is as short as 1 ms. Similarly at the thalamocortical synapse, one finds that individual afferents can evoke both excitation and feed-forward inhibition in the cortical cell, again separated by as little as 1 ms [18]. Because these delays are considerably shorter than the membrane time constant, the excitatory and inhibitory currents interfere effectively, as required for the simple tempotron model. For some cortical neurons, the inhibition from a sudden-onset stimulus even precedes the excitation, which allows a gating of responses with high temporal precision [66]. Thus there is precedent in cortical circuits for strong and rapid inhibition, including leading inhibition as also occurs in some of our examples (Figures 2A and 4E).

### Implications for Visual Processing Downstream of the Retina

What would be the identifying characteristics of neurons operating in the way we propose? At a minimum, the tempotron should have low background firing, so that individual spikes are significant events. It should respond reliably to a flashed or saccadic stimulus. And the very first spike of the response should already exhibit feature selectivity. Indeed, such neurons have been observed in V1 of the awake primates, with low maintained firing, and sharply tuned orientation selectivity in the earliest part of the response [67].

A downstream neuron dedicated to spike-latency processing would benefit from strong synaptic depression at its inputs (Figure 3B) to focus the computation on the first spike in the train. This could be an interesting marker of single-spike computations. Note that other postsynaptic neurons may observe the same spike train with non-depressing synapses, thus making use of slower components of the response. As applied to the visual processing after a saccade, one can envision a rapid feedforward sweep through dedicated cortical pathways that support the earliest appearance of object recognition, followed by a longer wave of activity that is shaped by recurrent and feedback processing [68] and subserves additional visual functions.

At the output end of the tempotron, the energetic cost of spikes would favor neurons that produce just one or zero spikes within a processing episode. Indeed, such binary responses have been observed in the auditory cortex, in response to brief tone presentations [69]. Because extracellular and optical recordings are biased against neurons with low spike numbers [62], detecting binary responses required the alternate approach of cell-attached recording [70]. Perhaps a similar search could be conducted in visual cortex, using natural saccade-fixation stimuli to which a rapid image-analysis pathway would be suited.

A more specific hallmark of our proposed mechanism is that it operates on the kinetic differences between On and Off channels arriving from the retina. As a consequence, the response latency of the tempotron depends on the nature of the visual task (Figures 6G and H). For example, the model cell of Figure 8 has identical orientation tuning for all grating phases, but its latency depends systematically on phase (Figure 8B). These predictions could be tested in neurophysiological experiments.

In this paper, we have focused on spike time computation in the visual system. But the model for spike-time computing proposed here is not specific to the processing of visual inputs. The exhibited power of the tempotron model suggests that it may be equally applicable to readout functions in other sensory systems – hearing, smell, touch, and electrosensation – where spike times have already been shown to convey important stimulus information [4], [71]–[76].

## Materials and Methods

### Ethics Statement

All experiments were carried out in accordance with the Guide for the Care and Use of Laboratory Animals of the National Institutes of Health, and this study was specifically approved by the Institutional Animal Care and Use Committee of Harvard University.

### Recordings

The experiments contributing to this study have been described in previous work [13]. In short, retinas were isolated from larval tiger salamanders, superfused with oxygenated Ringer’s medium at room temperature, and placed ganglion cell layer down on a multi-electrode array, which recorded spike trains from many ganglion cells simultaneously [77]. Spikes were sorted off-line by a cluster analysis of their shapes, and spike times were measured relative to the beginning of each stimulus repeat. Only units corresponding to well-separated spike clusters with a clear refractory period were included in further analysis. For these cells, the spike patterns during the 150-ms time window of stimulus presentation provided the input to the tempotron and perceptron models.

### Stimulation

Visual images were projected onto the photoreceptor layer of the retina via a CRT monitor with a frame rate of 66 Hz. White light was used with an average image luminance of 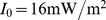 and a spectrum as described in [78]. For the salamander’s red cone photoreceptor, this mean intensity was equivalent to 20000 photons/µm^2^/s at 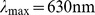. A gray screen of average luminance was displayed for 750 ms, followed by a square-wave grating for 150 ms. The bar width of the grating was 330 µm on the retina, somewhat larger than a typical ganglion-cell receptive field center. The eight different grating versions were obtained by successively shifting the grating by one fourth of the bar width. The minimal shift between gratings was 82.5 µm, considerably smaller than most ganglion-cell receptive field centers. The light and dark bars had intensity values 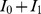 and 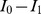, respectively, and the quoted contrast values are Michelson contrast, 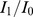. The gratings were presented in pseudo-random order, mixing the spatial phases of the grating as well as the contrast levels in experiments with varying contrast.

### Cell Types

To determine the cell type of the recorded retinal ganglion cells (RGCs), we analyzed the shape of the spike-triggered average recorded under a white-noise flicker stimulus and found different cell types according to a cluster analysis [13]. Here we focused exclusively on cells of the “fast Off”-type, which are characterized by an Off-type spike-triggered average but generally respond at both the onset and offset of a light step. This RGC type displays a pronounced latency code [13]. In total, the present work is based on 41 fast Off cells that were recorded in nine separate experiments with 8, 7, 5, 5, 5, 3, 3, 3, and 2 simultaneously recorded cells, respectively. From these, the eight-cell and two of the five-cell experiments were conducted with only the highest contrast level, yielding a total of 1658 trials. In the other experiments, several different contrast levels were used with roughly 280 trials per contrast condition.

### Characterization of Response Tuning

We characterized the first-spike latency and the spike count tuning of each RGC (Figure 1) by fitting a cosine tuning function of the form 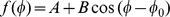 to each of the two response measures at a given stimulus contrast level. Here 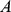 denotes the baseline of the response, 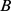 its modulation amplitude and 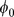 the phase offset. For the first-spike latency tuning, only trials with at least one spike were used in the fitting procedure. Fits and goodness-of-fit statistics were computed with the fit() function of the MATLAB Curve Fitting Toolbox environment. The coefficient of determination 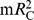 for mean spike counts was computed by using the fit parameters obtained with the single-trial spike counts to compute the expected counts 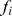 for each stimulus phase *i* and evaluating
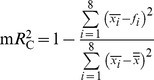
where the 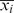 are the observed mean spike counts for each stimulus phase and 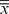 is the mean spike count over all phases. Coefficients of determination for the mean first-spike latencies 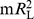 were computed analogously. This quantity corresponds to the fraction of variance explained by the fit.

### Tempotron Model

To read out the measured ganglion cell spike trains, we used the tempotron neuron model, an integrate-and-fire model together with a synaptic learning rule described previously [14]. Briefly, the sub-threshold voltage of the current-based leaky integrate-and-fire neuron model was given by a weighted sum of postsynaptic potentials (PSPs) from all incoming spikes:
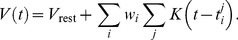
(1)


Here 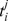 denotes the *j* ^th^ spike time of the *i* ^th^ afferent and
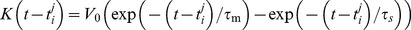
(2)is the normalized PSP contributed by each incoming spike. The factor 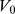 normalizes the peak amplitude to unity such that individual PSP amplitudes are given by the synaptic efficacies 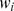. Except when stated otherwise, the time constant of membrane integration was set to 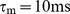. The decay time constant of synaptic currents was always 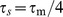, providing for a biologically realistic, fixed shape of the post-synaptic currents. The tempotron was trained to perform a given visual classification task by feeding the retinal population responses elicited by the corresponding visual stimuli as inputs into the tempotron and applying the tempotron learning rule: Following an error trial each synaptic efficacy 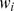 was modified by
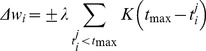
(3)with the change being positive if the neuron failed to spike during a target input spike pattern and negative if the neuron fired erroneously in response to a null input pattern [14]. Here 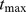 denotes the time at which the postsynaptic voltage 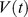 reaches its maximal value. The constant 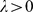 specifies the maximal size of the synaptic update per input spike. To accelerate learning, we used a momentum heuristic for the tempotron learning rule with a momentum parameter of 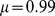 [14], [39].

### Tempotron with Depressing Synapses

Short-term synaptic dynamics (Figure 3B) were implemented following the model of Tsodyks et al. [21]. The static amplitude of postsynaptic potentials 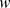 is scaled by the product of a depression variable that captures the depletion of synaptic resources due to previous spikes and a facilitation variable that mimics the spike-dependent dynamics of the release probability. For the present purpose we treat only depression [79]. The postsynaptic voltage (cf. Equation 1) is given by
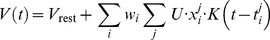
(4)where 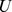 is the baseline efficacy of the synapse, and 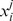 is a dynamic factor indicating depression of the *i* ^th^ afferent at its *j* ^th^ spike time, determined by
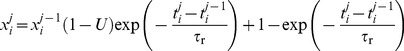
(5)and the initial condition 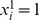. The ensuing synaptic dynamics are controlled by the baseline efficacy 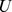, and the recovery time constant 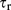. To accommodate this dynamic synaptic model, the tempotron learning rule for updating the synaptic weights was modified accordingly to
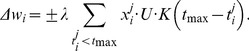
(6)


### Training Procedure and Performance Measurements

To analyze the ability of a neuronal decoder model to use a given retinal representation for visual processing, we studied two visual discrimination tasks (Figure 2B). For each task, two classes, consisting of two visual stimuli each, were selected, and the task for the tempotron was to detect one stimulus class by firing at least one spike while remaining silent for the other class. Given the eight stimulus gratings used in the present experiments, the two tasks could be realized in eight and four different ways, respectively, because of the different ways by which individual stimuli could be grouped into the stimulus classes. The reported performance measures are averages over all possible realizations of each task. For each task realization, we performed several learning runs. For each learning run, a readout neuron model was trained by cycling through all the relevant stimulus trials and applying the learning rules of the tempotron [14] or the perceptron [39], respectively. Learning started with random Gaussian initial synaptic efficacies (with zero mean and a range of standard deviations 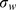 as specified below) and extended over 2000 cycles. The fraction of misclassified input patterns in each cycle was smoothed by a moving average that extended over 50 consecutive learning cycles, and the performance of a particular run was defined as the minimum of the resulting smoothed error curve.

Based on these individual learning runs, the performance of each neuron model for a particular task realization was defined as the minimal error that was achieved over 100 runs at each combination of the learning rule parameters 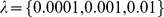 and 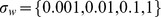. To study the tempotron’s visual processing capabilities in varying stimulus contrast conditions, we analyzed its performance on spike trains of a 7-cell population across 4 contrast levels. Here, the above training procedure was performed either with spike patterns from all 4 contrasts or with only the highest and lowest contrast conditions. The synaptic efficacies of the best readout neuron during this training were then used to measure its performance in each single contrast condition separately.

### Validation of Analysis Results

We probed the validity of the tempotron’s classification performance in the most general condition, i.e. with all ganglion cell spikes admitted for decoding and dynamic synapses (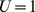; 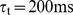), in two ways. First, we tested whether the performance extends to other RGC populations of similar size, by resampling many subsets of cells from our total pool of RGCs of this type. Specifically, the analysis was repeated over 10 randomly sampled populations of 8 RGCs out of the total of 18 RGCs that were measured in the high contrast condition and 10 populations of 7 RGCs sampled from the total of 23 RGCs measured in the variable contrast experiments. The average performance of these virtual populations matched the results obtained for the native populations on both tasks, with only a slight decrement of ∼2% on the boundary task. Second, we ruled out overfitting of the model by using separate subsets of the data for training (75%) and testing (25%). When using a training margin, optimized over (0, 0.025, 0.05, 0.1, 0.15, 0.2), this cross-validation produced essentially identical performance measures as the ones obtained with our above measure (the reduction in fraction correct of the tempotron was less than 0.07% for the luminance task and approximately 1.25% for the boundary task). This is expected because the dimensionality of the data vastly exceeds the number of parameters of the tempotron model.

### Temporal Winner-take-all Decoder

We compared the obtained classification results to the performance of a temporal winner-take-all model (Figure 3D). A binary temporal winner-take-all classifier [24] is fully characterized by labeling each afferent of an input population with one of the two possible classification decisions. For an incoming spike pattern among the afferents, the label belonging to the afferent with the shortest latency determines the decision of the classifier. In addition to this first-spike-based winner-take-all decoder, we also evaluated the performance of an extended winner-take-all decoder [25] whose decision was implemented as a majority vote between the afferents belonging to the three shortest latencies. For each task, both temporal-winner-take-all decoders were optimized over the entire data set, by an exhaustive search through all possible labelings of the afferents.

### Rank-order-based Decoder

For further comparison, we implemented a rank-order-based decoder (Figure 3D) following Delorme and Thorpe [26]. Briefly, this decoder is an integrate-and-fire neuron whose afferents each produce at most one spike. The post-synaptic potential is given by the sum 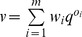 over all 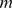 activated synapses, where 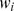 is the synaptic weight of the *i* ^th^ afferent, 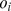 denotes the temporal rank of the *i* ^th^ afferent’s spike latency, and 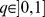 is an attenuation factor by which the neuron desensitizes after each spike. To make the decoder selective for a particular target condition, the synaptic efficacy of each afferent was set to its average attenuation factor over all the firing patterns in the target condition, 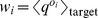 [26]. To obtain a conservative comparison, we optimized both the value of 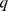 and the neuron’s firing threshold.

### Analysis of Retinal Ganglion Cell Pairs

To explore the basic computations underlying the tempotron decoding of spike-latency-based neuronal representations, we evaluated the tempotron’s classification performances on the basis of pairwise retinal inputs. The performance values for pairs of RGCs were based on exhaustive searches of the tempotron and perceptron parameter spaces. In these analyses, the input to the tempotron model was based on only the first spike of each ganglion cell. For each pair of ganglion cells, the reported performance refers to the best performance over all realizations of a given task type. This performance measure was chosen because the two receptive fields may, for some task realizations, fall outside the stimulus regions most relevant for the task.

To interpret relative spike latencies, the tempotron with just two inputs requires at least one spike per afferent. However, on some trials, especially at low stimulus contrast, certain ganglion cells failed to fire entirely. The quoted analyses of errors incorporate all trials, including such spike failures. To evaluate the speed of the tempotron decoding, we evaluated its operation under the constraint that only first spikes of each afferent with arrival times before a maximal time 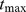 were used. The synaptic weights were optimized separately for each value of 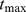.

We analyzed the robustness of the tempotron decoding to noise (Figure 6F) by fitting the input distribution of relative latencies with a Gaussian for each stimulus grating of the task realization. Then, for each value of 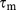, the tempotron’s weights were optimized such that the corresponding projection of the input distribution to maximal voltages yielded a minimal classification error when assuming a Gaussian threshold noise with zero mean and a standard deviation set to 5% of the mean weight magnitude.

### Analytical Treatment of the Tempotron with Two Afferents

To obtain a full understanding of the decoding of ganglion cell pairs we derived an analytic solution of the corresponding tempotron decoder (Figure 5). We considered a tempotron that is driven by two afferents with non-zero synaptic efficacies 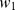 and 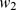, each firing exactly one spike per trial. The neuron maps each relative latency 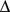 between these two input spikes into a peak postsynaptic voltage 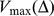. Importantly, this mapping is non-monotonic. If the magnitude of 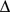 is large, the two inputs act essentially in isolation and 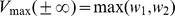 assumes the value of the larger of the two synaptic efficacies. We assume here that at least one of them is excitatory and their sum positive. If, on the other hand, the two input spikes arrive in synchrony, 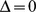, then 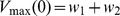 becomes the sum of the two.

Assuming that the neuron’s firing threshold lies between 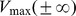 and 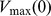, two behaviors emerge: Firstly, if both efficacies are excitatory 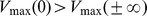, the neuron fires within a region of small 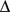 and remains silent if the magnitude of 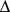 is large. Secondly, if one synapse is excitatory and the other inhibitory, the neurons fires if the magnitude of 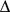 is large, but not if it is small. Hence, the tempotron with two afferents generates a tripartite segmentation of the space of relative latencies. The boundary between the different response regions is characterized by a pair of relative latencies, one for each firing order. Defining 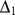 as the boundary for inputs with 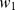 firing before 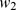 and 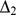 as the boundary when 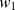 fires after 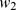, the maximal voltages obey 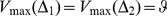, where 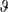 is the neuron’s firing threshold. Using analytical expressions for 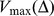, we numerically solved this equation for 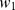 and 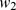.

### Model for Phase-invariant Orientation Tuning

To test whether a single tempotron can realize phase-invariant orientation tuning on the basis of realistic first-spike latency patterns, we trained the tempotron to respond to spike trains from a modeled retinal patch of 200 ganglion cells that was stimulated with square gratings of continuous spatial phase and orientation (Figures 7–8). For the description of the model’s configuration below, the spatial period of the stimulus grating is defined as 1. The 200 model ganglion cells had receptive fields with a Gaussian sensitivity profile, whose centers were randomly placed within a circular region of radius 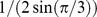. The standard deviation of each two-dimensional Gaussian receptive field was 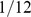. For a given grating stimulus with phase 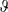 and orientation 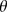, the first spike latency of the 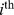 cell was given by
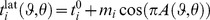
(7)where 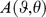 denotes the integral of the receptive field portion that is covered by dark areas of the grating and the offset 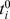 and modulation 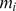 were drawn from normal distributions fitted to the latency statistics of our empirical ganglion cell sample (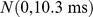
for 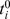 and 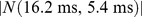 for 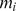).

Tempotrons were trained on a fine rectangular grid of 201 phases, which were linearly spaced between −180° and 180°, and 101 orientations, spanning the range between −90° and 90°. To obtain examples of wide and narrow tuning of orientation selectivity, the target stimuli consisted of all stimuli with orientations either between −15° and 15° (narrow tuning) or between −30° and 30° (wide tuning). Robust generalization beyond the training grid was ensured by training with a margin of ±10% of the firing threshold for all orientations, except near the boundary of the response region (within ±3° or ±6° for the 30°- or 60°-wide region, respectively). Initial synaptic weights were drawn from a Gaussian distribution with zero mean and a standard deviation of 0.001. To enhance the learning speed, we employed a schedule for the step size 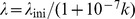 where 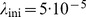 and 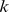 counts the number of presented input spike patterns. A momentum parameter of 0.99 was used. Learning continued for 10,000 cycles of the entire phase–orientation grid presented at a random but fixed order. With these parameters, 14 out of 20 tempotrons converged to zero error on the grid for the narrow tuning task and 15 out of 20 for the wide tuning task.
